# Pantothenic Acid Protects Neurons After Ischemic Stroke by Targeting ID3 to Restore Action Potential Amplitude

**DOI:** 10.1002/jnr.70130

**Published:** 2026-05-14

**Authors:** Hongqiao Chen, Mingli Chen, Lian Meng, Xing Wei, Yan Qin, Zhumei Bi

**Affiliations:** ^1^ Department of Neurology The First Affiliated Hospital of Guangxi University of Science and Technology Liuzhou City Guangxi Zhuang Autonomous Region China; ^2^ Department of Neurology The First Affiliated Hospital of Guangxi Medical University Nanning City Guangxi Zhuang Autonomous Region China

**Keywords:** electrophysiology, inhibitor of DNA binding 3, ischemic stroke, neuroprotection, pantothenic acid

## Abstract

Ischemic stroke (IS) remains a devastating condition with limited neuroprotective options. This study investigated the role of the transcription factor inhibitor of DNA binding 3 (ID3) in acute IS through an integrated approach. Combining bioinformatic analysis of Gene Expression Omnibus (GEO) datasets with machine learning (ML) algorithms, we identified ID3 as a consistently downregulated key gene, and its expression level correlated with neurological severity. Functional analysis suggested ID3 modulates neuroinflammation. Furthermore, ID3 and C‐type lectin domain family 4 member E (CLEC4E) showed potential as diagnostic biomarkers. Using network pharmacology, pantothenic acid (PA) was predicted as a potential ID3‐targeting drug. This was preliminarily tested in an oxygen–glucose deprivation/reperfusion (OGD/R) model, where PA treatment specifically upregulated ID3, ameliorated neuronal electrophysiological dysfunction, and restored action potential amplitude. Our work provides the first integrative evidence suggesting ID3 as a pivotal protective factor in acute IS and nominates PA as a candidate for further development as a neuroprotective agent.

## Significance Statement

- Ischemic stroke (IS) is a devastating neurological disorder and an unmet medical need that requires the identification of novel therapeutic targets. Our study demonstrated that pantothenic acid (PA), a widely available nutrient, protected neurons after ischemic injury by targeting ID3 and restored action potential amplitude.
- These results provide new evidence for PA application as a neuroprotective agent to treat ischemic stroke.
- Moreover, they pave the way to test PA in other neurological diseases characterized by neuronal injury and electrophysiological dysfunction.

## Graphical abstract

Ischemic stroke reduces ID3 expression and impairs neuronal action potential amplitude and frequency. Pantothenic acid (PA) targets and upregulates ID3, restoring amplitude but not frequency. The PA‐ID3‐amplitude axis represents a potential neuroprotective strategy.

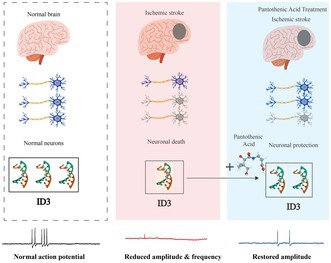

## 1 Introduction

Ischemic stroke (IS) represents a major global health burden. As the predominant form of stroke, it accounts for 5.2% of all deaths worldwide and is a leading cause of disability and cognitive impairment (Wang et al. 2022). IS triggers brain tissue death and focal neuronal injury, resulting in severe neurological deficits, high mortality rates, and long‐term functional impairments that significantly affect patients' quality of life (Wang et al. 2022). Current treatment options remain considerably limited. Most therapeutic agents suffer from poor delivery efficiency and a narrow therapeutic time window, leading to suboptimal outcomes and unfavorable prognoses (He et al. 2021). This therapeutic gap underscores the urgent need to develop novel strategies, such as neuroprotective agents and therapies targeting specific molecular pathways, to mitigate ischemic brain damage and improve recovery (Li, Liu, et al. 2023).

The core pathophysiological mechanism of ischemic injury is the “ischemic cascade,” which involves multiple processes including energy failure, calcium overload, oxidative stress, excitatory amino acid toxicity, and neuroinflammation, ultimately leading to neuronal death (Li, Chen, et al. 2025; Endres et al. 2022). Although current reperfusion therapies—such as intravenous thrombolysis and mechanical thrombectomy—are central to acute‐phase management and aim to rapidly restore blood flow to salvage the ischemic penumbra, they are constrained by strict time windows, bleeding risks, and technical accessibility. Moreover, the reperfusion process itself can cause reperfusion injury, exacerbating cerebral edema and hemorrhagic transformation (Ho and Powers 2025; Staessens and De Meyer 2020). Therefore, identifying adjuvant strategies that can interfere with the ischemic cascade, directly protect neurons, and improve neurological outcomes—particularly neuroprotective treatments following recanalization—has become a major focus and challenge in the field.

In recent years, researchers have been dedicated to identifying key molecular targets involved in ischemic stroke. Among these, inhibitor of DNA binding 3 (ID3), an important transcriptional regulator, has been found to participate in various biological processes including cell proliferation, differentiation, and survival (Chen et al. 2018). The brain mounts an endogenous neurogenic response after IS, and ID3—as a key regulator of neural stem/progenitor cell proliferation and differentiation—may influence post‐stroke neuro‐regenerative potential through its expression dynamics (Leggieri et al. 2020). Additionally, vascular remodeling is crucial during the recovery phase after IS, and ID3, which promotes vascular smooth muscle cell proliferation, may contribute to this restorative process (Wassmann et al. 2009). However, although the potential role of ID3 in the recovery phase has been widely discussed, its specific function and regulatory mechanisms in acute neuronal injury following IS remain poorly understood. To address this gap, we hypothesize that ID3 may play a more critical and direct role in the pathogenesis of IS. Yet, the expression dynamics of ID3 in the acute phase of IS and its direct role in neuronal damage have not been fully elucidated. Furthermore, whether ID3 can be targeted pharmacologically to exert neuroprotective effects remains an open question. Therefore, this study aims to investigate the potential role of ID3 in acute IS by: (1) exploring the expression pattern of ID3 through integrated bioinformatic analysis and preliminary validation with clinical samples; (2) screening for potential neuroprotective agents that may target ID3 using network pharmacology approaches; and (3) evaluating in an in vitro model whether the candidate drug can alleviate neuronal injury and improve electrophysiological function, and if so, whether this effect is associated with the regulation of ID3. This exploratory work seeks to provide novel insights and preliminary evidence for the development of future neuroprotective strategies against ischemic stroke. Through this integrative exploratory approach, we aim to provide the first evidence linking ID3 to acute ischemic neuronal injury and to establish it as a potential therapeutic target for neuroprotection.

## 2 Methods and Materials

### 2.1 Data Collection and Preprocessing

Datasets (GSE16561 and GSE58294) were obtained from the Gene Expression Omnibus (GEO) database. Following the methodology described by Li et al., the gene expression matrices were merged with corresponding annotation files for preprocessing (Li, Long, et al. 2023; Li, Zhu, et al. 2025; Li, Sun, et al. 2025). The two datasets were subsequently integrated and normalized using the R package “sva”. All data analyses were performed using the R programming language (version 4.3.2).

### 2.2 Identification and Comprehensive Analysis of Differentially Expressed Genes in IS

Volcano plots were created using the “limma” R package to identify differentially expressed genes (DEGs) in IS. The gene ontology (GO) functional enrichment analysis comprises three fundamental terms: biological process, cellular component, and molecular function (MF). The Kyoto encyclopedia of genes and genomes (KEGG) enrichment analysis focuses primarily on enriched pathways. We conducted GO and KEGG analyses to comprehensively evaluate the MFs of the DEGs.

### 2.3 Machine Learning‐Based Identification of Optimal DEGs

In this study, we implemented three distinct machine learning (ML) algorithms—Support Vector Machine (SVM), Random Forest (RF), and Least Absolute Shrinkage and Selection Operator (LASSO) regression—using R to identify differentially expressed genes in an Ischemic Stroke (IS) cohort. The SVM model was trained using the “e1071” package, while the RF algorithm was executed via the “randomForest” package. For LASSO regression, we utilized the “glmnet” package to perform feature selection with L1 regularization. The important feature sets extracted from each of these three models were subsequently integrated, and a consensus set of key differential genes was obtained by taking their intersection using base R functions.

### 2.4 SHAP‐Based Interpretation of Optimal DEGs in IS

To enhance the interpretability of our machine learning‐derived differential gene signatures, we implemented SHapley Additive exPlanations (SHAP) analysis for quantifying feature contributions. This analysis was performed using the SHAPforxgboost and fastshap packages in R (version 4.3.2), in combination with our previously trained XGBoost model built upon the identified optimal DEGs. The analytical process involved the following steps: First, we retrained an XGBoost classifier using the xgboost package with the consensus DEG set as input features. The model was configured with maximum depth = 5, eta = 0.01, and nrounds = 1000 through 10‐fold cross‐validation. Subsequently, we computed SHAP values using the shap.values() function from the SHAPforxgboost package, which provided both global feature importance and individual sample‐level explanations. For visualization, we generated summary plots displaying the distribution of SHAP values across all samples using shap.summary.plot(), and additionally created dependency plots for top‐ranked genes to reveal their directional relationships with ischemic stroke outcomes through shap.dependence.plot().

### 2.5 Correlation Analysis Between CRs and Infiltrated Immune Cells

Flow cytometry is commonly employed to investigate cellular heterogeneity; however, it imposes stringent requirements on sample preservation, as improper handling may lead to cell loss or damage, ultimately compromising data accuracy (Shen‐Orr and Gaujoux 2013). An alternative computational approach, Aaron M. Newman et al. developed CIBERSORT (Cell‐type Identification By Estimating Relative Subsets of RNA Transcripts), which infers the relative abundances of specific cell types from bulk gene expression data without being affected by physical sample degradation (Newman et al. 2015). Owing to its robustness against artifacts related to cell preservation, CIBERSORT has been widely adopted. In this study, we applied the CIBERSORT algorithm (accessible at https://CIBERSORT.stanford.edu/) along with the LM22 signature matrix to estimate the proportions of 22 immune cell subtypes within each sample based on gene expression profiles from the merged dataset. The immune subsets included memory B cells, naïve B cells, activated and resting dendritic cells, eosinophils, M0‐, M1‐, and M2‐macrophages, activated and resting mast cells, monocytes, neutrophils, activated and resting NK cells, plasma cells, activated and resting memory CD4 T cells, naïve CD4 T cells, CD8 T cells, follicular helper T cells, and gamma delta T cells. To further explore the relationship between differentially expressed circular RNAs (DE‐CRs) and immune microenvironment features, we performed correlation analysis using Spearman's rank correlation method. Associations with a *p*‐value < 0.05 were considered statistically significant.

### 2.6 Network Pharmacological Analysis

A network pharmacology approach was employed to further explore potential therapeutic mechanisms. Gene and drug data were obtained from the Drug Signatures Database (DSigDB). The analysis was conducted using R packages including “clusterProfiler”, “org.Hs.eg.db”, “enrichplot”, “tidytable”, and “ggplot2”. Resulting networks were visualized with Cytoscape (version 3.8.0). Additionally, molecular structures of the compounds in SDF format were retrieved from PubChem, and protein structures in PDB format were acquired from the RCSB Protein Data Bank. Cavity‐detection guided blind docking was subsequently performed to predict binding interactions between the selected ligands and target proteins.

### 2.7 Verifying the mRNA Expression of DEGs in Patients With IS

To preliminarily verify the bioinformatic predictions, a set of clinical blood samples was collected. Given the exploratory nature of this initial verification phase, a limited cohort consisting of six blood samples from age‐ and sex‐matched healthy individuals and six from patients with acute ischemic stroke (IS) was enrolled. The sample size was determined based on feasibility and was intended for initial trend observation rather than definitive statistical inference. The diagnostic criteria for IS were based on previous guideline (European Stroke Organization Executive Committee, and ESOW Writing Committee., 2008). The exclusion criteria were as follows: Hemorrhagic stroke, malignant tumor, metabolic disorders, infectious disease, pneumonia, and severe impairment of consciousness. Informed consent was obtained from patients or their guardians. This study adhered to the principles of the Declaration of Helsinki. Ethical approval was granted by the Institutional Review Board of The First Affiliated Hospital of Guangxi University of Science and Technology (No. KY‐LW‐001).

Total RNA was extracted from whole blood samples using RNAiso Blood reagent (9113; Takara). RNA concentration and purity were assessed spectrophotometrically (e.g., A260/A280 ratio between 1.8 and 2.0). Complementary DNA (cDNA) was synthesized from 1 μg of total RNA using the HiScript III 1st Strand cDNA Synthesis Kit (R312‐01, Vazyme) according to the manufacturer's protocol. qRT‐PCR was performed on a 7500 Real‐Time PCR System (Applied Biosystems) using Taq Pro Universal SYBR qPCR Master Mix (Q712‐02, Biosharp). The reaction mixture (20 μL total volume) contained 10 μL of master mix, 0.4 μL of each forward and reverse primer (10 μM), 2 μL of cDNA template, and 7.2 μL of nuclease‐free water. The thermal cycling conditions were as follows: initial denaturation at 95°C for 30 s, followed by 40 cycles of 95°C for 5 s and 60°C for 30 s. Melting curve analysis was performed to confirm primer specificity. Each sample was run in triplicate. The cycle threshold (Ct) values were normalized to the endogenous reference gene GAPDH (B661104; Sangon Biotech), and relative gene expression was calculated using the 2 − ΔΔCt method. The primers used for detecting DEGs were as follows: ID3 forward: 5′‐GCGAAGGACTGTGAACTTGTGG‐3′, reverse: 5′‐CACGCTCTGAAGAGACCTTAGAAC‐3′; CLEC4E forward: 5′‐ATTCATTCTCCCTGAATCTTACCAA‐3′, reverse: 5′‐GTATTGGTAGAATTTTATGCAATAATG‐3′.

### 2.8 Construction of the In Vitro Model of IS

The oxygen–glucose deprivation/reperfusion (OGD/R) model is widely used as an in vitro simulation of ischemic stroke (IS). In this study, cortical neurons isolated from 24‐h‐old Sprague–Dawley (SD) rats (obtained from the Animal Experiment Center of Guangxi Medical University) were used to establish an OGD/R model, following neuronal culture procedures adapted from Li, Chen, et al. (2025), Li, Sun, et al. (2025), Li, He, et al. (2025), Li, Wei, et al. (2023), Li, Zheng, et al. (2023), Zhu et al. (2024, 2025).

Prior to cell plating, 35‐mm culture dishes were coated with poly‐D‐lysine (Gibco, A3890401) and incubated at 37°C for 10 h. Two types of media were prepared: (1) Plating medium: 88% DMEM/F12 (Gibco, A4192001) supplemented with 1% GlutaMAX (Gibco, 35050061), 1% penicillin–streptomycin (Gibco, 15140‐122), and 10% fetal bovine serum (Gibco, A3160902); (2) Maintenance medium: 96% Neurobasal‐A medium (Gibco, 10888022) supplemented with 1% GlutaMAX, 1% penicillin–streptomycin, and 2% B‐27 supplement (Gibco, 17504044). The cerebral cortex was dissected from anesthetized newborn Sprague–Dawley rats (within 24 h of birth) in ice‐cold Hank's Balanced Salt Solution (HBSS, ThermoFisher, #14185052) containing 10 mM HEPES buffer. The isolated cortical tissue was then minced and digested with 0.125% trypsin–EDTA (Gibco, A4192001) at 37°C for 12 min. The enzymatic reaction was stopped by adding an equal volume of the plating medium. After trituration, the cell suspension was passed through a 40‐μm nylon mesh cell strainer to remove undigested tissue fragments. The dissociated cortical neurons were counted using a hemocytometer and plated onto the coated dishes at a density of 2 × 10^5^ cells/dish in 2 mL of plating medium. After allowing the cells to adhere for 10 h, the plating medium was completely replaced with the serum‐free maintenance medium. Half of the maintenance medium was refreshed every 3 days thereafter.

All experiments involving rats were performed in accordance with the IACUC and NIH guidelines. After 8 days in vitro (DIV8), the cultured neurons were assigned to either control or OGD/R groups. The detailed protocol was performed as described by Lai et al. (2020). Neurons in the control group were maintained in complete culture medium under standard conditions. For the OGD/R group, cells were rinsed three times with phosphate‐buffered saline (PBS) and transferred to an anaerobic chamber containing a glucose‐free Dulbecco's Modified Eagle Medium (DMEM; Gibco, Cat# 11966‐025, NY, USA) under an atmosphere of 5% CO_2_ and 95% N_2_ at 37°C for 2 h. Following the deprivation phase, the medium was replaced with normal culture medium, and the neurons were returned to a normoxic incubator (5% CO_2_, 37°C) for 12 h to simulate reperfusion injury.

The experimental protocol utilizes an 8‐concentration gradient of pantothenic acid (PA) (0, 1, 5, 10, 20, 50, 100, and 200 μM) applied to in vitro neurons for 24 h: 0–5 μM was designed to model the pathological deficiency state observed in Lewy body dementia brains (20%–40% reduction in regions like the hippocampus) and align with human serum physiological baselines (1–2 μM; Scholefield et al. 2024); 10–20 μM reflects the effective concentration range for pantothenate pathway modulation demonstrated in fungal models (5–20 μM; Choi et al. 2024); 50–200 μM incorporates tolerance evidence from engineered bacteria synthesizing high‐concentration pantothenate (0.44 M) and biocompatibility data from vitamin B5‐derived lipid delivery systems (Song et al. 2024; Yoo et al. 2024); the 24‐h exposure duration is supported by rapid CoA‐dependent metabolic responses (confirmed in fungal systems within this timeframe) and acute neuronal energy impairment models in neurodegenerative pathology.

### 2.9 Electrophysiological Recordings

The electrophysiological recordings were performed following established protocols described by Li, Wei, et al. (2023), Li, Zheng, et al. (2023), Zhu et al. (2024, 2025). Action potentials (APs) were acquired in whole‐cell patch‐clamp configuration using a Digidata 1550B amplifier and a 16‐bit Axon Digidata 1550B acquisition system controlled by pClamp 10.7 software. Cells were bathed in an extracellular solution with the following composition (in mM): 122 NaCl, 2 KCl, 25 HEPES, 2 CaCl_2_, 4 MgCl_2_, and 10 glucose (pH adjusted to 7.4). Patch pipettes (3–6 MΩ resistance) were filled with an internal solution containing (in mM): 110 KCl, 1 NaCl, 2 EGTA, 25 HEPES, 4 Mg‐ATP, 0.3 Na_2_‐GTP, and 10 phosphocreatine (pH 7.3). All recordings were conducted at a holding potential of −70 mV in the whole‐cell mode.

### 2.10 Construction of the in Vivo Model of IS

The middle cerebral artery occlusion (MCAO) model is considered an in vivo model of IS (Lai et al. 2020). Sixteen adult male Sprague–Dawley rats (weight 200–250 g) were randomly allocated into two groups: sham operation (*n* = 8) and MCAO (*n* = 8). All animals were housed under standard conditions (12‐h light/dark cycle, 22°C ± 2°C, 50% ± 10% humidity) with free access to food and water. The experimental protocol was approved by the Animal Ethics Committee of Guangxi Medical University (ethical batch number: No. 202403125), and all procedures were conducted in accordance with the National Institutes of Health Guide for the Care and Use of Laboratory Animals.

Anesthesia and Surgical Preparation: Rats were anesthetized via inhalation of 5% isoflurane in an induction chamber and maintained with 2%–2.5% isoflurane delivered through a nose cone. The depth of anesthesia was monitored by the absence of the pedal withdrawal reflex. After the loss of consciousness, the cervical region was shaved and disinfected with alternating applications of 75% ethanol and povidone‐iodine.

MCAO Procedure: A midline neck incision was made. Under an operating microscope, the right common carotid artery (CCA), external carotid artery (ECA), and internal carotid artery (ICA) were carefully exposed and isolated. Body temperature was monitored via a rectal probe and maintained at 37.0°C ± 0.5°C using a homeothermic heating pad throughout the surgery. In the MCAO group, the CCA and ICA were temporarily clamped using microvascular clips. A silicon‐coated nylon monofilament (diameter: 0.26 ± 0.02 mm; MEYUE, M8509) was introduced through a small incision in the ECA and gently advanced into the ICA until a mild resistance was felt (approximately 18–20 mm from the bifurcation), indicating occlusion at the origin of the middle cerebral artery (MCA). The filament was left in place for 90 min to induce ischemia. Sham‐operated rats underwent identical procedures, including vessel exposure and clamping, but the filament was not advanced to occlude the MCA.

Postoperative Care and Sample Collection: Following surgery, the filament was carefully withdrawn to allow reperfusion, and the incision was sutured. Rats were placed in a warm, clean cage to recover from anesthesia and were monitored daily for neurological deficits and overall health. Buprenorphine (0.05 mg/kg, subcutaneously) was administered postoperatively for analgesia. Neurological function was assessed 24 h after reperfusion by an investigator blinded to the group allocation, using a standardized scoring system: 0, no deficit; 1, failure to fully extend the left forelimb; 2, circling to the left; 3, falling to the left; 4, no spontaneous walking.

Seven days after surgery, rats were euthanized for tissue collection. Euthanasia was performed under deep anesthesia (5% isoflurane) by transcardial perfusion (i.e., perfusion via the heart) with ice‐cold phosphate‐buffered saline (PBS), followed by rapid decapitation. For the three rats randomly selected from each group for Triphenyl tetrazolium chloride (TTC) staining, brains were immediately removed. The remaining five rats per group were used for molecular biology experiments, and brain tissues from the ipsilateral (ischemic) and contralateral hemispheres were rapidly dissected on ice, snap‐frozen in liquid nitrogen, and stored at −80°C until analysis.

### 2.11 Triphenyl Tetrazolium Chloride (TTC) Staining

After anesthesia, the rats were sacrificed, and two rats from each group were randomly selected for TTC staining. The brain tissue was collected and placed directly into the optimal cutting temperature compound embedding agent for quick freezing at −20°C for 30 min. Then, the brain was sliced, and each slice was approximately 2–3 mm thick. The TTC solution was preheated to 37°C. Then, the slices were incubated with TTC solution at 37°C for 30 min in the dark. The tissues were gently shaken for 10 min to ensure equal exposure to the dye solution, and the staining results were observed.

### 2.12 Western Blot

Proteins were extracted using a commercial kit (Invent Biotechnologies, SD‐001/SN‐002; Li, Wei, et al. 2023). The following primary antibodies were employed: anti‐ID3 (Sangon Biotech, D199808; dilution 1:200), anti‐CLEC4E (Santa, sc‐390806; dilution 1:500), anti‐NeuN (Abcam, ab236870; dilution 1:1000), and anti‐GAPDH (Abcam, ab8245; dilution 1:10,000). Protein bands were visualized using an Odyssey Infrared Imaging System (LI‐COR Biosciences). Quantification of target proteins was performed with ImageJ software, with all signals normalized to the internal control GAPDH. Each experiment was repeated three times independently.

### 2.13 Neuronal Transfection

Lentivirus (LV) was used as the vector to intervene in the ID3 expression of neurons. Neurons at DIV3 were divided into the negative control group (NC) and the ID3 overexpression group (OE). The lentiviruses were purchased from Sangon Biotech (Shanghai) Co. Ltd., including negative control (NC, titer: 3.83 × 10^9^ TU/mL) and ID3 overexpression (OE, titer: 3.27 × 10^9^ TU/mL). Based on previous study (Li, Wei, et al. 2023), lentiviruses were added to antibiotic‐free medium at a multiplicity of infection (MOI) = 5 and incubated with the neurons. After 24 h, the medium was replaced with normal neuronal maintenance medium. On DIV8, the neurons were subjected to subsequent experiments.

### 2.14 Statistical Analysis

All data are presented as mean ± standard deviation. The normality of data distribution was assessed using the Shapiro–Wilk test. For data that followed a normal distribution, comparisons between two groups were performed using unpaired two‐tailed Student's *t*‐test. Comparisons among multiple groups were analyzed using one‐way analysis of variance (ANOVA), followed by post hoc tests (Dunnett's or Tukey's method, as specified in the figure legends based on the experimental design). For data that violated the normality assumption, the non‐parametric Mann–Whitney *U* test was used for two‐group comparisons. For comparisons among more than two groups, the Kruskal–Wallis *H* test was employed. If the Kruskal–Wallis test indicated a statistically significant difference, Dunn's post hoc test was subsequently performed for pairwise comparisons. Correlation analysis was conducted using Spearman's rank correlation. All statistical analyses were carried out with SPSS software (version 25.0). A *p*‐value < 0.05 was considered statistically significant. For analyses involving multiple comparisons (e.g., multiple electrophysiological parameters), appropriate corrections (such as the Bonferroni correction) were applied. A completed transparency checklist following the Journal of Neuroscience Research guidelines is provided in the Supporting Information.

## 3 Results

### 3.1 Identify DEGs in the IS Population and the Biological Functions of These DEGs

**FIGURE 1:**
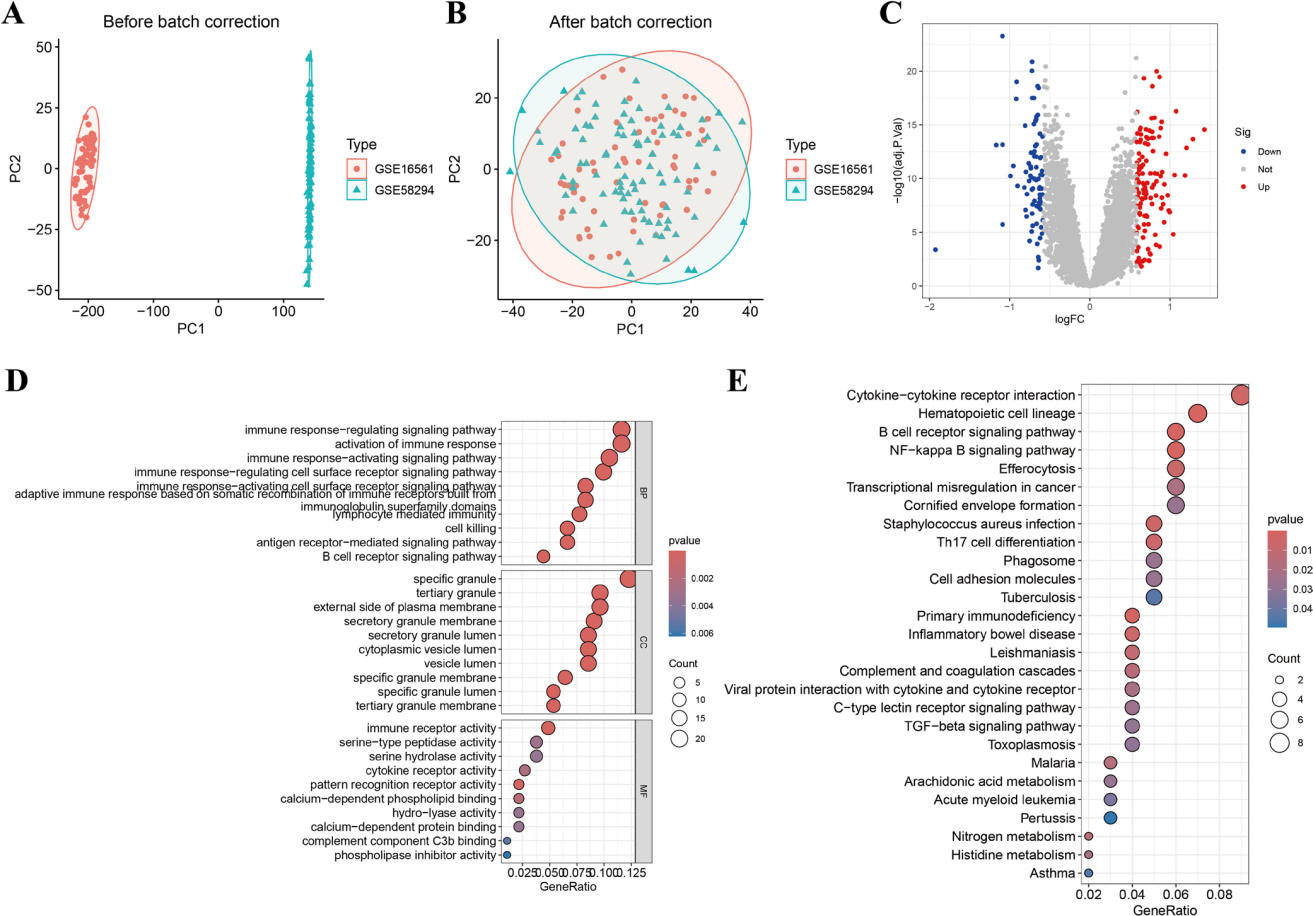
Identification of differentially expressed genes (DEGs) and their functional enrichment in IS. (A, B) Batch effect correction and integration of two independent gene expression datasets (GSE16561 and GEG58294). (C) Volcano plot showing significantly upregulated (red) and downregulated (blue) DEGs in IS compared to controls. (E) Significantly enriched Gene Ontology (GO) terms and Kyoto Encyclopedia of Genes and Genomes (KEGG) pathways among the DEGs, highlighting involvement in immune and inflammatory responses.

We integrated and performed batch correction on two distinct datasets, achieving satisfactory results that support further investigation (Figure 1A,B). Differential expression analysis was conducted on the merged dataset, and the differentially expressed genes (DEGs) are displayed in a volcano plot (Figure 1C). Subsequent biological functional analysis of the DEGs revealed significant enrichment in IS patients for GO terms such as immune response‐regulating signaling pathway, activation of immune response, activating signaling pathway, and regulation/activation of cell surface receptor signaling pathway (Figure 1E). KEGG pathway analysis indicated enrichment in IS patients for pathways including Cytokine‐cytokine receptor interaction, Hematopoietic cell lineage, B cell receptor signaling pathway, NF‐kappa B signaling pathway, and Efferocytosis (Figure 1E).

### 3.2 Value of ID3 and CLEC4E in Predicting IS Risk

**FIGURE 2:**
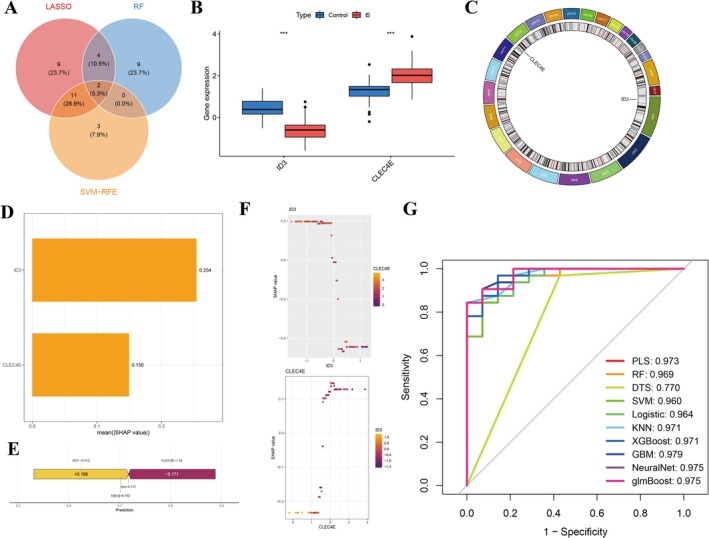
Machine learning‐based identification of candidate genes and evaluation of their diagnostic value. (A) Venn diagram showing the overlap of feature genes selected by LASSO, SVM, and Random Forest algorithms. (B) Expression levels of ID3 and CLEC4E in IS and control groups. (C) Genomic locations of ID3 and CLEC4E. (D) Contribution of ID3 and CLEC4E features in the predictive model. (G) Receiver operating characteristic (ROC) curve demonstrating the performance of the ID3 + CLEC4E‐based classifier in predicting IS risk.

An intersection analysis using LASSO, RF, and SVM models identified two overlapping genes (Figure 2A). Among the IS population, ID3 expression was significantly downregulated, while CLEC4E expression was upregulated (Figure 2B). Chromosomal locations of ID3 and CLEC4E are illustrated in Figure 2C. The contribution values of ID3 and CLEC4E to the predictive model are shown in Figure 2D. The features “ID3 = −0.512” and “CLEC4E = 1.54” exhibited positive contributions to the IS prediction model, with SHAP values of 0.186 and −0.171, respectively. The ROC curve and AUC of the model constructed based on the expression levels of ID3 and CLEC4E for predicting IS risk are presented in Figure 2G.

### 3.3 Immune Cell Infiltration Characteristics in IS

**FIGURE 3:**
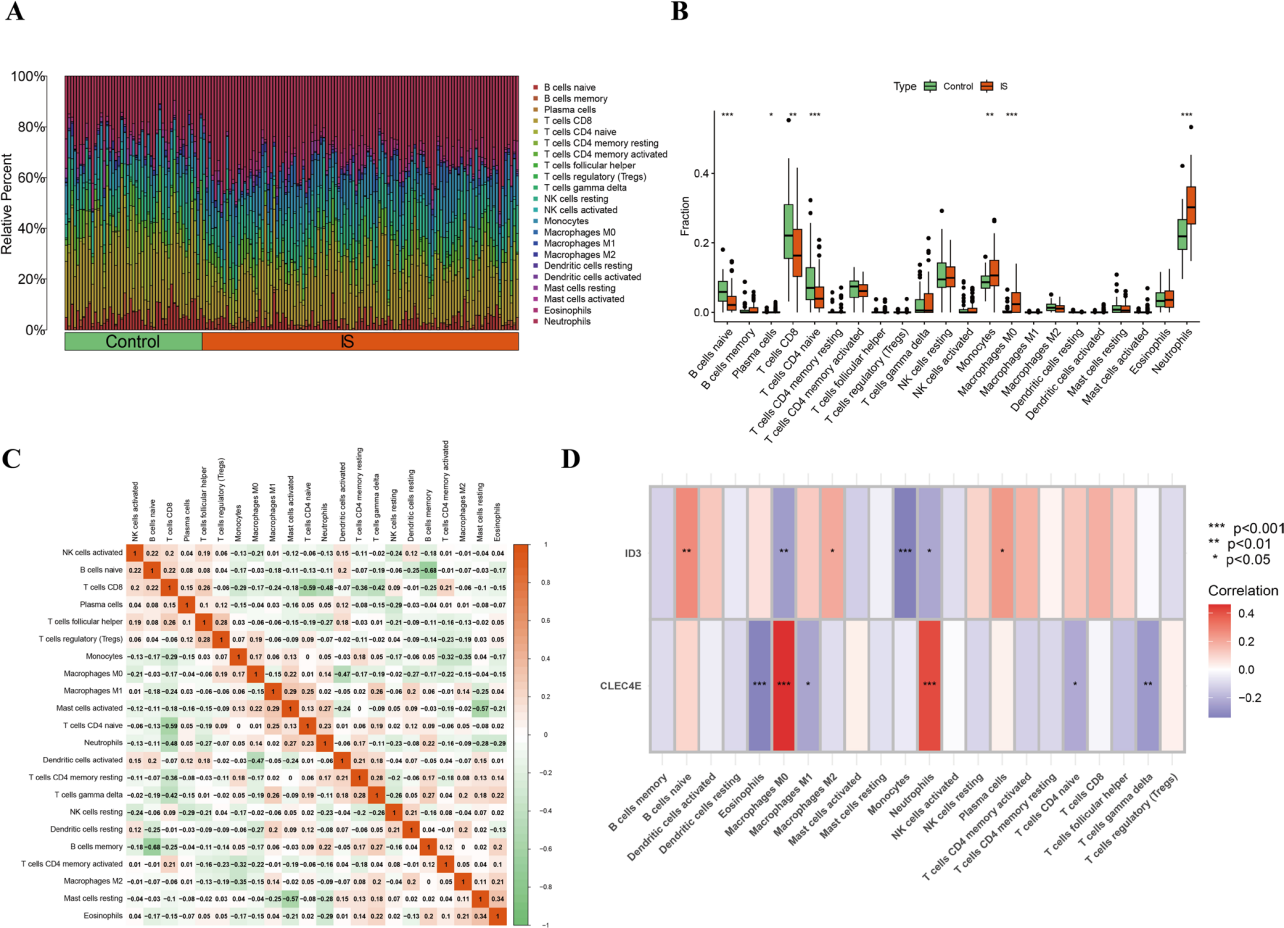
Immune infiltration landscape and correlation with key genes in IS. (A) Relative abundance of 22 immune cell types in IS and control samples, estimated by CIBERSORT. (B) Differential immune cell infiltration between groups. (C) Correlation network among immune cell subtypes. (D) Spearman correlation analysis between ID3/CLEC4E expression and immune cell proportions.

The abundance of immune cells in patients from the Ctrl and IS groups is shown in Figure 3A. Differences in immune cell infiltration between the Ctrl and IS groups are displayed in Figure 3B. B cells naïve, T cells CD8, and T cells CD4 naïve were significantly decreased in IS patients, whereas plasma cells, monocytes, macrophages M0, and neutrophils were significantly increased. Correlations among various immune cell types are illustrated in Figure 3C. ID3 showed positive regulatory correlations with B cells naïve, macrophages M2, and plasma cells, and negative correlations with neutrophils, macrophages M0, and monocytes (Figure 3D). CLEC4E was positively correlated with macrophages M0 and neutrophils, and negatively correlated with eosinophils, macrophages M1, T cells CD4 naïve, and T cells gamma delta (Figure 3D).

### 3.4 PA Targets ID3 to Restore Neuronal Action Potentials and Protect Neurons After IS

**FIGURE 4:**
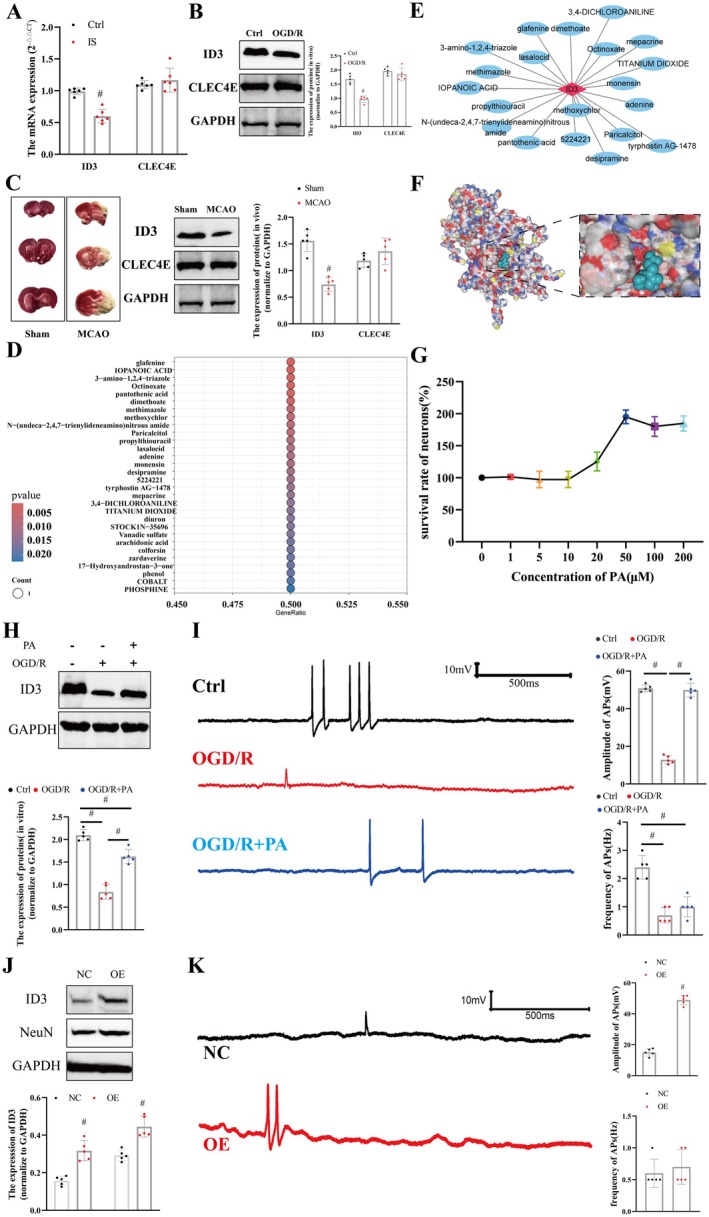
Experimental validation of ID3 downregulation and neuroprotective effect of PA via targeting ID3. (A) ID3 mRNA expression in clinical blood samples from IS patients and controls (*n* = 6). (B) ID3 expression in cortical neurons after oxygen–glucose deprivation/reperfusion (OGD/R) injury (*n* = 5). (C) TTC staining of brain sections from MCAO model rats (upper), and ID3 expression in brain tissue (lower) (*n* = 5). (D) Drug‐target interaction network based on ID3. (E) Drug‐target interaction network based on ID3. (F) Molecular docking simulation between ID3 and PA. (G) Neuronal viability under different concentrations of PA after OGD/R. (H) ID3 expression levels after treatment with 50 μM PA (*n* = 5). (I) Representative traces and quantitative analysis of action potential amplitude and frequency in neurons from Control, OGD/R, and OGD/*R* + PA groups (*n* = 5). (J) ID3 and NeuN expression after ID3 overexpression. The expression of ID3 and NeuN was significantly elevated in the OE group (n=5). (K) Representative traces and quantitative analysis of action potential amplitude and frequency in neurons from NC and OE groups (n=5). Data are presented as mean ± SD; # *p* < 0.01.

Analysis of mRNA expression in clinical blood samples revealed significantly reduced ID3 expression in the IS group (*n* = 6 per group; Ctrl vs. IS, *p* = 0.000011; Student's *t*‐test; Figure 4A), while CLEC4E expression showed no significant change (*n* = 6 per group; Ctrl vs. IS, *p* = 0.39, Student's *t*‐test; Figure 4A). In the OGD/R group, ID3 expression was decreased (*n* = 5 per group; Ctrl vs. OGD/R, *p* = 0.000039, Student's *t*‐test; Figure 4B), though CLEC4E expression remained unchanged (*n* = 5 per group; Ctrl vs. OGD/R, *p* = 0.36, Student's *t*‐test; Figure 4B). TTC staining showed significant ischemic damage in brain tissues of the MCAO group (Figure 4C), indicating successful model establishment. ID3 expression was also reduced in the MCAO group (*n* = 5 per group; Ctrl vs. MCAO, *p* = 0.000064, Student's *t*‐test; Figure 4C), with no significant change in CLEC4E expression (*n* = 5 per group; Ctrl vs. MCAO, *p* = 0.19, Student's *t*‐test; Figure 4C). Based on ID3, potential targeted drugs were screened, with significance illustrated in Figure 4D. A network pharmacology diagram of ID3 and its targeted drugs is presented in Figure 4E. The molecular docking between ID3 and PA is shown in Figure 4F. We then evaluated the neuroprotective effects of different concentrations of PA on OGD/R‐treated neurons. Results indicated that 50 μM PA yielded the optimal neuronal survival rate (Figure 4G). After treatment with 50 μM PA, ID3 expression in neurons was restored and showed upregulation compared to the OGD/R group (*n* = 5 per group; Ctrl vs. OGD/*R* + PA, *p* = 0.002645, Tukey's method; OGD/R vs. OGD/*R* + PA, *p* = 0.000159, Tukey's method; Figure 4H). Finally, we examined neuronal action potentials (APs). Results demonstrated that the amplitude (*n* = 5 per group; Ctrl vs. OGD/R, *p* = 7.3159E‐9, Tukey's method; Figure 4I) of APs was significantly reduced in the OGD/R group, but PA treatment restored AP amplitude (*n* = 5 per group; Ctrl vs. OGD/*R* + PA, *p* = *p* = 0.003, Tukey's method; Figure 4I). The frequency of APs differed significantly among the three groups (Kruskal–Wallis test, *H* = 10.706818, *p* = 0.004732; Figure 4I). Dunn's post hoc comparison revealed that the frequency was significantly lower in the OGD/R group compared to the Ctrl group (*p* = 0.0046; Figure 4I), whereas no significant difference was observed between the OGD/R + PA and OGD/R groups (*p* > 0.99; Figure 4I). Finally, we employed LV transduction in neurons and established the OGD/R model. In the OE group, ID3 expression was significantly elevated (*n* = 5 per group; NC vs. OE, *p* = 0.000074, Student's *t*‐test; Figure 4J). Concurrently, the expression of NeuN, a neuronal marker, also increased (*n* = 5 per group; NC vs. OE, *p* = 0.000542, Student's *t*‐test; Figure 4J). Electrophysiological results indicated that in the OE group, the amplitude of APs was significantly increased (*n* = 5 per group; NC vs. OE, *p* = 4.6824E‐8, Student's *t*‐test; Figure 4K), whereas the frequency showed no significant change (*n* = 5 per group; NC vs. OE, *p* = 0.54, Student's *t*‐test; Figure 4K).

## 4 Discussion

This study through an integrative exploratory approach provides first evidence suggesting the critical protective role of ID3 in the acute phase of ischemic stroke (IS) through integrated bioinformatic analysis, machine learning algorithms, and experimental validation. Our preliminary data demonstrated consistent downregulation of ID3 in peripheral blood from IS patients, as well as in both oxygen–glucose deprivation/reperfusion (OGD/R) and middle cerebral artery occlusion (MCAO) models, with its expression level closely correlated with the extent of neurological impairment. Furthermore, using network pharmacology screening and molecular docking, we identified pantothenic acid (PA) as a potential targeted activator of ID3. Subsequently, we performed in vitro experiments to preliminarily test that PA specifically upregulates ID3 expression, significantly ameliorates OGD/R‐induced electrophysiological dysfunction in neurons, and restores action potential amplitude. These findings suggested the potential importance of ID3 as a novel molecular marker and therapeutic target in acute IS injury but also provide a basis for future investigation and translational exploration for developing ID3‐centered neuroprotective strategies.

The central role of ID3 in the pathogenesis of IS was robustly supported by multi‐level data. We first observed significant downregulation of ID3 expression in clinical blood samples and the rat MCAO model, suggesting that its loss is closely associated with acute ischemic events and may serve as a sensitive biological indicator of neuronal injury. Notably, ID3 also showed consistently low expression in the OGD/R in vitro model, further supporting its direct regulatory function in neuronal ischemic stress. Bioinformatic analysis provided important clues to the underlying mechanisms of ID3: GO and KEGG enrichment analyses revealed that differentially expressed genes were significantly enriched in biological processes such as immune response‐regulating signaling pathways, cell activation, and receptor signal transduction—consistent with previous studies confirming the key roles of these pathways in the pathogenesis of IS, even promoting disease progression and exacerbating neuronal damage (Kato et al. 2024). On the other hand, immune infiltration analysis indicated that ID3 expression positively correlated with protective immune subsets such as naïve B cells and CD4^+^ T cells, while showing significant negative correlations with pro‐inflammatory M0 macrophages, monocytes, and neutrophils. Previous studies have reported that naïve B cells and CD4^+^ T cells can suppress inflammatory responses and alleviate neural damage (Chien et al. 2025; Guy et al. 2023), whereas M0 macrophages, monocytes, and neutrophils may exacerbate tissue damage through the release of inflammatory mediators (Chen et al. 2023; Shi et al. 2022). These results collectively suggest that ID3 may exert a protective role in the ischemic cascade by modulating neuroinflammatory responses and immune microenvironment homeostasis, and its decreased expression could lead to exacerbated immune dysregulation and neuronal injury.

To reliably identify feature genes with diagnostic value, this study employed an integrated multi‐machine‐learning algorithm strategy, identifying key genes ID3 and CLEC4E through the intersection of three models: LASSO regression, random forest (RF), and support vector machine (SVM). This approach effectively reduced the risk of overfitting inherent in single‐model methods, improved the specificity of gene selection, and enhanced the robustness of the results, thereby establishing a rigorous computational biology foundation for subsequent biomarker development (Li, Sun, et al. 2025; Chen et al. 2025). It is noteworthy that ID3 and CLEC4E not only exhibited significant differential expression (downregulation of ID3 and upregulation of CLEC4E) but, more importantly, formed a diagnostic combination with potential clinical application value. SHAP analysis further quantified the contributions of these two genes to the predictive model: ID3 had a SHAP value of 0.186, indicating a positive risk contribution, while CLEC4E had a SHAP value of −0.171, indicating a negative contribution. This antagonistic direction of effect is highly consistent with their expression changes in IS, enhancing their interpretability and reliability as biological markers. Particularly notable is the excellent discriminatory performance of the predictive model constructed based on these two genes, as evidenced by a high area under the ROC curve (AUC), further highlighting the future translational potential, which requires validation in larger cohorts, of the ID3 and CLEC4E combination as an auxiliary diagnostic or risk prediction tool for IS (Li, Zhu, et al. 2025).

Among the candidate genes screened by machine learning, we selected ID3 as the focus for subsequent in‐depth mechanistic exploration and drug targeting research for several reasons. First, compared to CLEC4E, ID3 showed more pronounced and consistent downregulation in IS patients, MCAO animal models, and OGD/R cellular models, suggesting that it may play a more critical regulatory role in acute ischemic injury. Second, previous literature widely reports that ID3 plays important roles in cell proliferation, differentiation, and survival, particularly exhibiting potential neuroprotective functions in the nervous system (Leggieri et al. 2020), while research on CLEC4E is largely confined to innate immunity and inflammatory responses (Pahari et al. 2019; Wodelo et al. 2024). Additionally, SHAP analysis indicated that ID3 has a relatively high contribution to the predictive model (SHAP value = 0.186), and its expression positively correlated with multiple protective immune cell subsets (e.g., naïve B cells and CD4^+^ T cells) and negatively correlated with pro‐inflammatory populations, suggesting that ID3 not only holds significance in the predictive model but may also occupy a central position in the regulation of the immune microenvironment. After establishing the central role of ID3, we further employed a network pharmacology approach to screen for targeted compounds, aiming to provide a theoretical basis and candidate drugs for subsequent intervention studies. By integrating multiple compound‐target interaction databases, we predicted PA as a potential modulator of ID3. Molecular docking simulations showed that PA binds to the ID3 protein with low binding energy and a stable conformation, suggesting strong affinity and specific interaction between the two (Qin and Tse 2025). This computational biology evidence provides a structural basis for considering PA as a potential targeted compound for ID3, successfully bridging bioinformatically identified targets with experimental pharmacology and laying a solid foundation for subsequent functional validation and mechanistic studies at the cellular and animal levels.

Previous clinical studies have reported that patients with cerebral infarction may exhibit electroencephalogram (EEG) abnormalities such as focal slow‐wave activity enhancement, disorganized brain rhythms, and decreased neuronal electrical activity frequency, reflecting impaired excitability and disrupted synchronization of neurons within the ischemic core and penumbra (Fukujima and Cardeal 1997; Ferreira et al. 2023). Furthermore, ischemia and hypoxia can lead to reduced neuronal action potential firing frequency, decreased amplitude, and impaired synaptic transmission efficacy, electrophysiological dysfunctions closely associated with the degree of neurological deficit in patients (Auer et al. 2016; Zheng et al. 2019). Against this clinical and experimental background, our study focused on the restorative effects of PA on post‐ischemic neuronal electrophysiological function. In the in vitro OGD/R model, we observed electrophysiological impairments corresponding to clinical EEG abnormalities: significant reductions in both the amplitude and frequency of neuronal action potentials. After PA intervention, not only was ID3 expression restored, but more importantly, neuronal electrical activity was markedly improved, manifested as a significant recovery of action potential amplitude. This suggests that PA may enhance ion channel function or membrane excitability in ischemic neurons by upregulating ID3 expression, thereby partially reversing the electrical suppression caused by ischemic damage. Thus, our findings functionally connect clinically observed EEG abnormalities to cellular electrophysiological dysfunctions recorded in the laboratory and further demonstrate the potential therapeutic value of the PA–ID3 pathway in restoring neuronal electrical activity. This not only provides electrophysiological evidence for understanding the neuroprotective mechanism of PA but also offers new experimental support for future stroke treatment strategies aimed at recovering neural electrical activity. However, we recognize that the neuroprotective effects of PA may not be exclusively mediated by ID3, and other pathways may also contribute.

Importantly, the causal role of ID3 as a key neuroprotective mediator was further solidified by our gain‐of‐function experiment. Lentivirus‐mediated overexpression of ID3 in cortical neurons, even in the absence of PA, was sufficient to significantly elevate the expression of the neuronal marker NeuN and, more critically, to enhance the amplitude of action potentials following OGD/R injury. This finding provides direct genetic evidence that upregulation of ID3 alone is capable of promoting neuronal survival and restoring a key aspect of electrophysiological function, thereby fulfilling a central criterion for establishing ID3 as a downstream effector. It is noteworthy that ID3 overexpression specifically rescued action potential amplitude—mirroring the effect of PA treatment—but did not alter its firing frequency, which further refines our mechanistic understanding. Action potential amplitude is primarily governed by the density and functional state of voltage‐gated sodium channels responsible for depolarization (Greer et al. 2012), whereas its frequency is finely tuned by pacemaker currents (e.g., specific calcium‐activated potassium channels) that control rhythmic activity (Jacobson et al. 2010). Therefore, our data suggested that the PA‐ID3 pathway may be preferentially coupled to restoring or stabilizing the function of ion channels governing the depolarization phase (e.g., sodium channels), rather than directly modulating the pacemaking mechanisms responsible for intrinsic firing patterns. Collectively, these converging data from pharmacological (PA) and genetic (OE‐ID3) interventions robustly support the hypothesis that ID3 is not merely a correlative marker but a functionally significant protective factor in ischemic neuronal injury, with its mechanism potentially specifically targeting the maintenance of neuronal depolarization capability.

This study has several limitations that should be acknowledged to properly frame its conclusions, which are primarily exploratory and hypothesis‐generating. First and foremost, while our data establish a strong correlative and restorative link, they fall short of proving a causal mechanism. The observation that PA treatment rescues both ID3 expression and neuronal function, while highly suggestive, does not definitively establish ID3 as the necessary mediator of PA's effects. It remains possible that PA acts through parallel or upstream pathways, with ID3 upregulation being a concomitant event rather than the central effector. Therefore, the primary future direction emanating from this work is the direct genetic validation of ID3's role. To unequivocally test the hypothesis that PA protects neurons specifically by upregulating ID3, loss‐of‐function and gain‐of‐function experiments are essential. Knocking down ID3 expression in cortical neurons using lentiviral shRNA prior to OGD/R and PA treatment would test its necessity: an attenuation of PA's neuroprotective effects would strongly support ID3 as a crucial mediator. Conversely, overexpressing ID3 in the absence of PA could test its sufficiency for protection. These experiments represent the critical next step to transform the compelling associations described here into a defined molecular pathway. Second, the clinical sample size was relatively small (*n* = 6). This inherently limits the statistical power and generalizability of the findings from the clinical cohort. Consequently, the clinical data should be considered preliminary and hypothesis‐generating. Subsequent expansion of the cohort and multi‐center validation are necessary to robustly assess the reliability of ID3 and CLEC4E as potential biomarkers. Third, the current findings are based mainly on in vitro cell models. Although we confirmed ID3 downregulation in the MCAO rat model, the in vivo therapeutic efficacy, pharmacokinetic properties, and long‐term functional recovery effects of PA have not been evaluated. Lastly, although the network pharmacology approach is systematic, direct biochemical validation of the physical interaction between PA and the ID3 protein is still needed. Despite these limitations, this study successfully identifies ID3 as a novel and promising target in acute IS and proposes a readily available nutraceutical, PA, as its candidate modulator. By clearly outlining the above mechanistic and translational research agenda, we aim to provide a solid foundation and a clear roadmap for future investigations aimed at harnessing the ID3 pathway for neuroprotection.

## 5 Conclusion

In summary, this study integratively explores the critical protective role of ID3 in ischemic stroke (IS) by integrating bioinformatics, machine learning, and experimental validation. We not only observed the consistent downregulation of ID3 in both IS patients and disease models but also revealed its close association with immune microenvironment homeostasis. Furthermore, we identified for the first time that pantothenic acid (PA) may serve as a neuroprotective agent targeting ID3. More importantly, using in vitro models, we demonstrated that PA treatment significantly reversed the oxygen–glucose deprivation/reperfusion (OGD/R)‐induced suppression of ID3 expression, effectively improved neuronal survival, and restored action potential amplitude, thereby partially rescuing the impaired electrophysiological function of neurons following ischemic injury. These findings propose a potential “PA–ID3–neuronal functional recovery” axis, thereby offering a novel perspective and a testable hypothesis for future research into IS pathology and therapeutic development.

## Author Contributions

Hongqiao Chen designed the implementation of the research, drafted the preliminary papers, performed the in vitro/in vivo model, and participated in the investigations. Mingli Chen performed the western blot. Lian Meng performed the TTC assay. Xing Wei performed the patch clamp. Yan Qin was responsible for data analysis and data visualization. Zhumei Bi participated in the research design and implementation, manuscript revision, and supervised the study. All the authors read and approved the final manuscript.

## Funding

This study was supported by grants from Guangxi Zhuang Autonomous Region Health Commission (Grant No. Z‐B20220932).

## Ethics Statement

This study adhered to the ethical principles of the Declaration of Helsinki. The protocol involving human subjects was approved by the Human Ethics Committee of The First Affiliated Hospital of Guangxi University of Science and Technology (No. KY‐LW‐001). All animal experiments were approved by the Ethics Committee of Guangxi Medical University (ethical batch number: No. 202403125) and conducted in accordance with the National Institutes of Health Guide for the Care and Use of Laboratory Animals.

## Consent

The authors have nothing to report.

## Conflicts of Interest

The authors declare no conflicts of interest.

## Supporting information


**Data S1:** Transparent‐Science‐Questionnaire.

## Data Availability

The publicly available datasets (GSE16561 and GSE58294) analyzed in this study can be found in the Gene Expression Omnibus (GEO) repository. The original experimental data supporting the findings of this study are available from the corresponding author upon reasonable request.

## References

[jnr70130-bib-0001] Auer, L. M. , G. Pfurtscheller , S. Abobaker , E. Ott , K.‐J. Marguc , and H. Lechner . 2016. “Penumbra Around Chronic Cerebral Infarction?” Neurological Research 10: 246–251. 10.1080/01616412.1988.11739850 2907114

[jnr70130-bib-0002] Chen, F. F. , X. Lv , Q. F. Zhao , et al. 2018. “Inhibitor of DNA Binding 3 Reverses Cisplatin Resistance in Human Lung Adenocarcinoma Cells by Regulating the PI3K/Akt Pathway.” Oncology Letters 16: 1634–1640. 30008847 10.3892/ol.2018.8849 PMC6036442

[jnr70130-bib-0003] Chen, W. , J. Miao , J. Chen , and J. Chen . 2025. “Development of Machine Learning Models for Diagnostic Biomarker Identification and Immune Cell Infiltration Analysis in PCOS.” Journal of Ovarian Research 18: 1. 39754246 10.1186/s13048-024-01583-1 PMC11697806

[jnr70130-bib-0004] Chen, X. , L. Cheng , Y. Pan , et al. 2023. “Different Immunological Mechanisms Between AQP4 Antibody‐Positive and MOG Antibody‐Positive Optic Neuritis Based on RNA Sequencing Analysis of Whole Blood.” Frontiers in Immunology 14: 1095966. 36969199 10.3389/fimmu.2023.1095966 PMC10036921

[jnr70130-bib-0005] Chien, C. H. , T. Y. Yeh , and B. L. Chiang . 2025. “Non‐Antigen‐Specific B Cells Induced Regulatory CD4+ T Cells Through Decreasing T Cell Activation.” Immunology 175: 434–443. 40320632 10.1111/imm.13940

[jnr70130-bib-0006] Choi, J.‐Y. , S. Gihaz , M. Munshi , et al. 2024. “Vitamin B5 Metabolism Is Essential for Vacuolar and Mitochondrial Functions and Drug Detoxification in Fungi.” Communications Biology 7: 894. 39043829 10.1038/s42003-024-06595-7 PMC11266677

[jnr70130-bib-0007] Endres, M. , M. A. Moro , C. H. Nolte , C. Dames , M. S. Buckwalter , and A. Meisel . 2022. “Immune Pathways in Etiology, Acute Phase, and Chronic Sequelae of Ischemic Stroke.” Circulation Research 130: 1167–1186. 35420915 10.1161/CIRCRESAHA.121.319994

[jnr70130-bib-0008] Ferreira, L. O. , R. D. de Souza , L. L. Teixeira , et al. 2023. “The GPER1 Agonist G1 Reduces Brain Injury and Improves the qEEG and Behavioral Outcome of Experimental Ischemic Stroke.” Journal of Neuropathology & Experimental Neurology 82: 787–797. 37558387 10.1093/jnen/nlad061

[jnr70130-bib-0009] Fukujima, M. M. , and J. O. Cardeal . 1997. “Subsidiary Predictive Tests in Epileptic Crisis After Ischemic Stroke.” Arquivos de Neuro‐Psiquiatria 55: 39–45. 9332559 10.1590/s0004-282x1997000100007

[jnr70130-bib-0010] Greer, J. E. , J. T. Povlishock , and K. M. Jacobs . 2012. “Electrophysiological Abnormalities in Both Axotomized and Nonaxotomized Pyramidal Neurons Following Mild Traumatic Brain Injury.” Journal of Neuroscience 32: 6682–6687. 22573690 10.1523/JNEUROSCI.0881-12.2012 PMC3705917

[jnr70130-bib-0011] Guy, T. V. , A. M. Terry , H. M. McGuire , E. Shklovskaya , and B. Fazekas de St Groth . 2023. “MHCII Restriction Demonstrates B Cells Have Very Limited Capacity to Activate Tumour‐Specific CD4 + T Cells In Vivo.” Oncoimmunology 13: 2290799. 38125720 10.1080/2162402X.2023.2290799 PMC10730170

[jnr70130-bib-0012] He, W. , Z. Zhang , and X. Sha . 2021. “Nanoparticles‐Mediated Emerging Approaches for Effective Treatment of Ischemic Stroke.” Biomaterials 277: 121111. 34488117 10.1016/j.biomaterials.2021.121111

[jnr70130-bib-0013] Ho, J. P. , and W. J. Powers . 2025. “Contemporary Management of Acute Ischemic Stroke.” Annual Review of Medicine 76: 417–429. 10.1146/annurev-med-050823-094312 39496213

[jnr70130-bib-0014] Jacobson, D. A. , F. Mendez , M. Thompson , J. Torres , O. Cochet , and L. H. Philipson . 2010. “Calcium‐Activated and Voltage‐Gated Potassium Channels of the Pancreatic Islet Impart Distinct and Complementary Roles During Secretagogue Induced Electrical Responses.” Journal of Physiology 588: 3525–3537. 20643768 10.1113/jphysiol.2010.190207 PMC2988516

[jnr70130-bib-0015] Kato, Y. , D. Aburakawa , R. Tashiro , et al. 2024. “Intravenous Administration of Muse Cells Improves Cerebral Ischemia Outcome via Immunomodulation in the Spleen.” Journal of Cerebral Blood Flow & Metabolism 45: 542–557. 39397400 10.1177/0271678X241290363 PMC11563515

[jnr70130-bib-0016] Lai, Y. , P. Lin , M. Chen , et al. 2020. “Restoration of L‐OPA1 Alleviates Acute Ischemic Stroke Injury in Rats via Inhibiting Neuronal Apoptosis and Preserving Mitochondrial Function.” Redox Biology 34: 34. 10.1016/j.redox.2020.101503 PMC7327985 32199783

[jnr70130-bib-0017] Leggieri, A. , A. Palladino , C. Attanasio , et al. 2020. “Id(Entifying) the Inhibitor of DNA Binding 3 in the Brain of *Nothobranchius furzeri* Upon Aging.” Journal of Anatomy 238: 1106–1115. 33314133 10.1111/joa.13367 PMC8053586

[jnr70130-bib-0018] Li, S. , N. Chen , J. He , X. Luo , and W. Lin . 2025. “NDUFA11 May Be the Disulfidptosis‐Related Biomarker of Ischemic Stroke Based on Integrated Bioinformatics, Clinical Samples, and Experimental Analyses.” Frontiers in Neuroscience 18: 1505493. 39877656 10.3389/fnins.2024.1505493 PMC11772302

[jnr70130-bib-0019] Li, S. , J. He , H. Kuang , et al. 2025. “Rab11a‐Dependent Recycling of Glut3 Inhibits Seizure‐Induced Neuronal Disulfidptosis by Alleviating Glucose Deficiency.” Cell & Bioscience 15: 69. 40437641 10.1186/s13578-025-01396-9 PMC12121293

[jnr70130-bib-0020] Li, S. , Q. Long , L. Nong , Y. Zheng , X. Meng , and Q. Zhu . 2023. “Identification of Immune Infiltration and Cuproptosis‐Related Molecular Clusters in Tuberculosis.” Frontiers in Immunology 14: 1205741. 37497230 10.3389/fimmu.2023.1205741 PMC10366538

[jnr70130-bib-0021] Li, S. , L. Sun , H. Huang , et al. 2025. “Identifying Disulfidptosis‐Related Biomarkers in Epilepsy Based on Integrated Bioinformatics and Experimental Analyses.” Neurobiology of Disease 205: 106789. 39805370 10.1016/j.nbd.2025.106789

[jnr70130-bib-0022] Li, S. , X. Wei , H. Huang , et al. 2023. “Neuroplastin Exerts Antiepileptic Effects Through Binding to the α1 Subunit of GABA Type A Receptors to Inhibit the Internalization of the Receptors.” Journal of Translational Medicine 21: 707. 37814294 10.1186/s12967-023-04596-4 PMC10563248

[jnr70130-bib-0023] Li, S. , Y. Zheng , Q. Long , et al. 2023. “Drug–Drug Interactions Between Propofol and ART Drugs: Inhibiting Neuronal Activity by Affecting Glucose Metabolism.” CNS Neuroscience & Therapeutics 30: e14437. 37650345 10.1111/cns.14437 PMC10916437

[jnr70130-bib-0024] Li, S. , Q. Zhu , A. Huang , et al. 2025. “A Machine Learning Model and Identification of Immune Infiltration for Chronic Obstructive Pulmonary Disease Based on Disulfidptosis‐Related Genes.” BMC Medical Genomics 18: 7. 39780155 10.1186/s12920-024-02076-2 PMC11715737

[jnr70130-bib-0025] Li, Z. , B. Liu , K. L. Lambertsen , et al. 2023. “USP25 Inhibits Neuroinflammatory Responses After Cerebral Ischemic Stroke by Deubiquitinating TAB2. Advanced.” Science 10: 10. 10.1002/advs.202301641 PMC10558664 37587766

[jnr70130-bib-0026] Newman, A. M. , C. L. Liu , M. R. Green , et al. 2015. “Robust Enumeration of Cell Subsets From Tissue Expression Profiles.” Nature Methods 12: 453–457. 25822800 10.1038/nmeth.3337 PMC4739640

[jnr70130-bib-0027] Pahari, S. , S. Negi , M. Aqdas , E. Arnett , L. S. Schlesinger , and J. N. Agrewala . 2019. “Induction of Autophagy Through CLEC4E in Combination With TLR4: An Innovative Strategy to Restrict the Survival of *Mycobacterium tuberculosis* .” Autophagy 16: 1021–1043. 31462144 10.1080/15548627.2019.1658436 PMC7469444

[jnr70130-bib-0028] Qin, J. , and W. K. F. Tse . 2025. “In Silico Analysis Identified Potential Interaction Between Glutathione and Spliceosome in Nager Syndrome.” Free Radical Biology and Medicine 239: 43–48. 40681060 10.1016/j.freeradbiomed.2025.07.026

[jnr70130-bib-0029] Scholefield, M. , S. J. Church , J. Xu , S. Patassini , and G. J. S. Cooper . 2024. “Localized Pantothenic Acid (Vitamin B5) Reductions Present Throughout the Dementia With Lewy Bodies Brain.” Journal of Parkinson's Disease 14: 965–976. 10.3233/JPD-240075 PMC11307062 38820022

[jnr70130-bib-0030] Shen‐Orr, S. S. , and R. Gaujoux . 2013. “Computational Deconvolution: Extracting Cell Type‐Specific Information From Heterogeneous Samples.” Current Opinion in Immunology 25: 571–578. 24148234 10.1016/j.coi.2013.09.015 PMC3874291

[jnr70130-bib-0031] Shi, B. , Y. Hao , W. Li , H. Dong , M. Xu , and P. Gao . 2022. “TIPE2 May Target the Nrf2/HO‐1 Pathway to Inhibit M1 Macrophage–Related Neutrophilic Inflammation in Asthma.” Frontiers in Immunology 13: 883885. 35572500 10.3389/fimmu.2022.883885 PMC9095941

[jnr70130-bib-0032] Song, F. , Z. Qin , K. Qiu , et al. 2024. “Development of a Vitamin B5 Hyperproducer in *Escherichia coli* by Multiple Metabolic Engineering.” Metabolic Engineering 84: 158–168. 38942195 10.1016/j.ymben.2024.06.006

[jnr70130-bib-0033] Staessens, S. , and S. F. De Meyer . 2020. “Thrombus Heterogeneity in Ischemic Stroke.” Platelets 32: 331–339. 32310709 10.1080/09537104.2020.1748586

[jnr70130-bib-0034] Wang, X. , B. Feng , Z. Huang , et al. 2022. “Relationship of Cumulative Exposure to the Triglyceride‐Glucose Index With Ischemic Stroke: A 9‐Year Prospective Study in the Kailuan Cohort.” Cardiovascular Diabetology 21: 66. 35505313 10.1186/s12933-022-01510-y PMC9066788

[jnr70130-bib-0035] Wassmann, K. , C. F. H. Mueller , U. M. Becher , et al. 2009. “Interaction of Inhibitor of DNA Binding 3 (Id3) With Gut‐Enriched Krüppel‐Like Factor (GKLF) and p53 Regulates Proliferation of Vascular Smooth Muscle Cells.” Molecular and Cellular Biochemistry 333: 33–39. 19618124 10.1007/s11010-009-0201-7

[jnr70130-bib-0036] Wodelo, W. , E. Wampande , A. Andama , D. Kateete , and K. Ssekatawa . 2024. “Polymorphisms in Immune Genes and Their Association With Tuberculosis Susceptibility: An Analysis of the African Population.” Application of Clinical Genetics 17: 33–46. 38567200 10.2147/TACG.S457395 PMC10986402

[jnr70130-bib-0037] Yoo, S. , M. Faisal , S. H. Bae , et al. 2024. “Novel Less Toxic, Lymphoid Tissue‐Targeted Lipid Nanoparticles Containing a Vitamin B5‐Derived Ionizable Lipid for mRNA Vaccine Delivery.” Advanced Healthcare Materials 14: e2403366. 39502027 10.1002/adhm.202403366 PMC11912100

[jnr70130-bib-0038] Zheng, Y. , Y. Wu , Y. Liu , et al. 2019. “Intrinsic Effects of Gold Nanoparticles on Oxygen–Glucose Deprivation/Reperfusion Injury in Rat Cortical Neurons.” Neurochemical Research 44: 1549–1566. 31093902 10.1007/s11064-019-02776-7

[jnr70130-bib-0039] Zhu, Q. , H. He , Q. Long , et al. 2025. “Lactate‐Dehydrogenase‐5 May Play a Key Role in the Disturbance of Brain Energy Caused by Tuberculous Meningitis.” Journal of Integrative Neuroscience 24: 26741. 40302261 10.31083/JIN26741

[jnr70130-bib-0040] Zhu, Q. , Q. Long , C. Wei , et al. 2024. “Lactate Dehydrogenase‐1 May Play a Key Role in the Brain Energy Disturbance Caused by Cryptococcal Meningitis.” Journal of Microbiology, Immunology and Infection 57: 887–895. 10.1016/j.jmii.2024.08.009 39214781

